# Potential utility of reflectance spectroscopy in understanding the paleoecology and depositional history of different fossils

**DOI:** 10.1038/s41598-020-73719-4

**Published:** 2020-10-08

**Authors:** Swagata Chaudhuri, Arindam Guha, Ajoy K. Bhaumik, Komal Pasricha

**Affiliations:** 1 Department of Applied Geology, Indian Institute of Technology (Indian School of Mines), Dhanbad, Jharkhand 826 004 India; 2 Geosciences Group, National Remote Sensing Centre, Indian Space Research Organisation, Balanagar, Hyderabad, 500037 India; 3 Ministry of Earth Science, Government of India, New Delhi, 110003 India

**Keywords:** Biogeochemistry, Palaeoceanography, Palaeoclimate, Palaeoecology, Marine biology, Palaeontology

## Abstract

The potential of reflectance spectroscopy to infer the paleoecological and depositional evolution of different micro and macro invertebrate fossils has been evaluated by analyzing their reflectance spectra within the spectral domain of 350–2500 nm using the FIELDSPEC3 spectroradiometer. Mineralogical information derived from the rapid and non-destructive spectral analysis has been substantiated using concurrent mineralogical data from conventional geochemical analyses. The diagnostic Fe-crystal field effect induced spectral features are identified on the representative spectra of different benthic foraminifera. These spectral features are resulted due to the incorporation of Fe during the biomineralization process. These features are absent in planktic foraminifera. The encrustation of Fe-oxides is inferred to be responsible for imprinting the Fe-crystal field feature in the spectra of micro and macrofossils at 900–1200 nm. Vibrational spectral features of the Al–OH bond are also identified. Both of these features are an indicator of post-depositional diagenetic history. The presence of Al and Fe in macrofossil shells is also believed to be related to ecological conditions as these elements are biogenically incorporated during shell formation. This study reveals the value of reflectance spectroscopy to infer ecological behavior and post-depositional environment of different organisms.

## Introduction

Reflectance spectroscopy deals with the mineralogical analysis of spectral features of natural targets imprinted on their reflectance spectra^1–5^. It is a rapid, non-destructive analytical technique, which provides information about the mineralogy of rocks or any other natural constituents. In the reflectance spectra of rocks and minerals, we find several absorption kinks, which are flanked with localized reflectance maxima^5,6^. These absorption kinks are known as spectral features or absorption features and wavelengths of these absorption features are indicative of different types of atomic processes^7^. Many of these atomic processes are guided by the transition of electrons within the atomic structure of the element of interest and vibration of molecular bonds within them^8^. The reflectance spectra of rocks/substances can be collected from any distance; therefore, it is also suitable for imaging surface mineralogical variations from remote platforms^7^. Reflectance spectroscopy has been widely used to spatially map rocks and minerals on different planets and also on the Earth’s surface as different minerals have different electronic and vibrational absorption features^9–17^. More often, these spectral contrasts are being upscaled to the imaging reflectance spectroscopic data collected by space-based sensors for remote mapping of rocks and minerals^6,7,18^. In spite of reflectance spectroscopy being an established method to differentiate various materials based on their structure and geochemistry, the use of reflectance spectroscopy for fossil investigation is not common. Some researchers suggest that remote sensing technology might be used to locate the fossiliferous zones^19,20^. Various authors have suggested that reflectance spectroscopy can be reliably used to delineate different carbonate mineral phases present in the fossiliferous rocks^21–23^. In this regard, Anemone et al.^24^ pursued their investigation using remote sensing data and implemented a neural network to identify the fossiliferous horizon in the Great Basin of the USA. It is established that foraminifera and other macro fauna (Mollusca and Brachiopoda) have carbonate hardcover (calcite or aragonite) along with some other elements/minerals incorporated during the test calcification depending upon their habitat^25–27^. Although different fossils have spectrally distinctive minerals like calcite, aragonite, dolomite, siderite, etc.^28–30^, until now, there is no record on the utilization of reflectance spectroscopy as a tool for fossil characterization. Further, other elements (Fe, Al etc.) and textural variations (such as agglutinated, hyaline, porcellanitic, microgranular) in the fossil shells produce spectral contrasts^30^. Reflectance spectroscopy may be used as a rapid, non-destructive analytical technique for the assessment of mineralogy of fossil shells to facilitate mineralogical analysis of intact fossil samples without affecting their morphology. Reflectance spectra of fossil shells can provide immediate information on the presence of different elements like Fe, Mn etc. in the carbonate shells of the fossils and also may provide a clue on their relative abundance^31–33^. Furthermore, spectral features imprinted on the reflectance spectra of fossil shells may be suitable to indicate the presence of aqueous fluid inclusions and their relative abundance in the shell^32^. Information on the relative quantity of aqueous solution in the fossil shells may provide new insights for diagenetic studies^32^. Moreover, reflectance spectroscopy being a non-contact analytical technique, the spectral information retrieved from the shells may be upscaled to infer the mineralogical variation in spatial domain based on the processing of imaging spectroscopy data (reflectance spectra collected as a two-dimensional image). This may help in relating mineralogy of fossil shells with the broader mineralogical variation of the basin if fossiliferous litho-units are mappable in the pixels of the imaging spectrometer data. Spectral features of intimate mixtures (like rock surface or fossil shell) are difficult to infer if the spectral data are not analyzed with reference to the mineralogical data^5^. Therefore, analysis of spectral features of reflectance spectra is often supplemented with mineralogical data^34^.

In this study, we have made an attempt to analyze the reflectance spectra of different benthic micro and macrofossils from fossiliferous horizons. The benthic forms selected for this study were found abundantly in the shelf regions^35,36^. Spectral features of benthic fossils (both micro and macro) are analyzed as these fossils are sensitive to the geochemical parameters of the depositional surface^35–38^. Therefore, the mineralogical analysis of benthic fossils may provide valuable input for understanding the depositional settings and related paleoecological conditions. In addition to benthic fossils, a few planktic microfossils (planktic foraminifera live within the photic zone i.e., top 200 m of the sea surface) and vagile mollusks, such as cephalopods, were also studied to analyze the mineralogical information of these fossil shells. This mineralogical information is helpful to understand the geochemistry of the depositional medium, i.e., sea water^39^. Therefore, combined mineralogical analyses of benthic and planktic forms provide valuable input for understanding the depositional settings and paleoenvironment (primarily governed by the mineralogy of depositional surface and geochemistry of seawater) of the shelf region as both these groups reflect ocean hydrographic characteristics^35,40^. Such a perspective of study is absent in the literature. The present study is aimed to bring out the potential of reflectance spectroscopy in capturing the primary shell characteristics to infer paleoecology and their relationship with depositional setup.

## General geology

**Figure 1:**
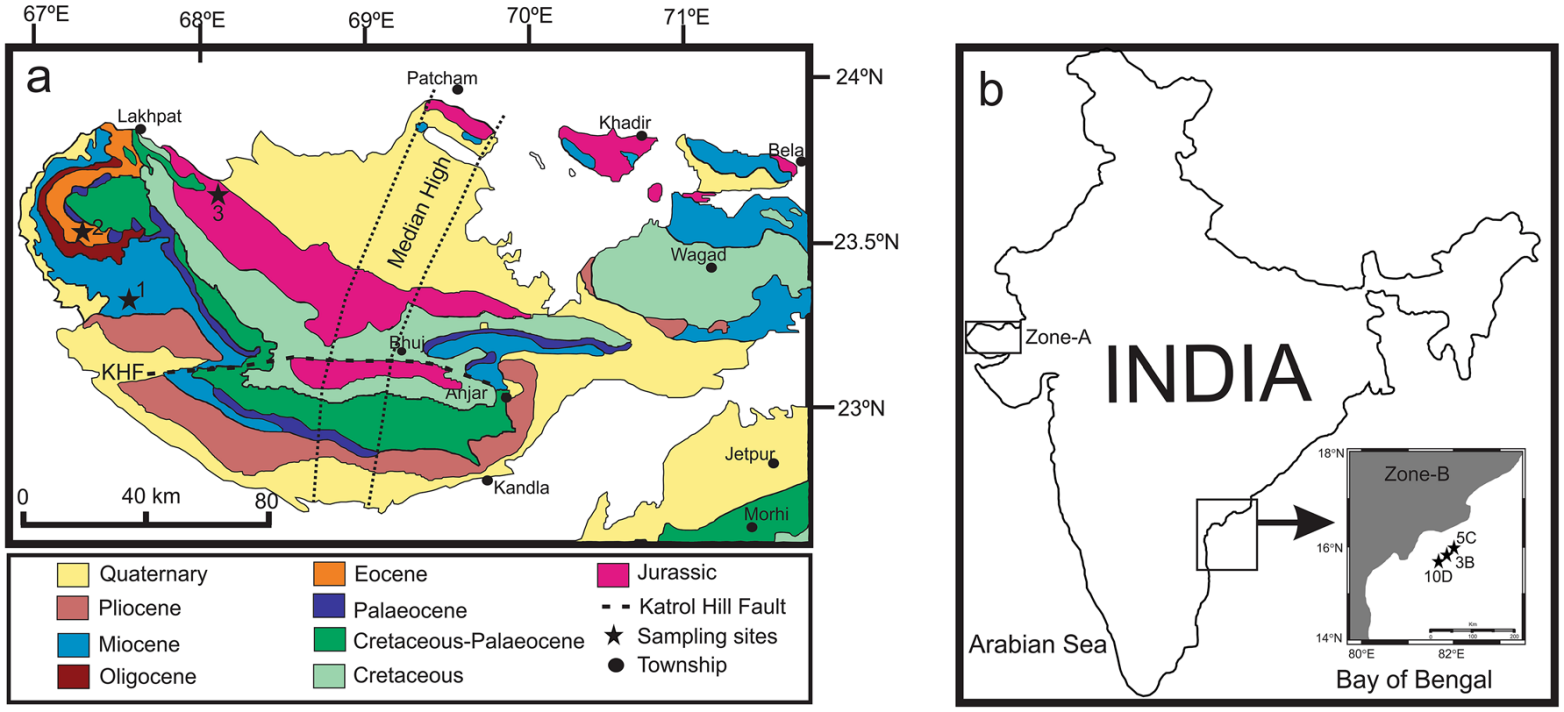
(**a**) Geological Map of Gujarat (Zone-A) showing the larger benthic foraminiferal and invertebrate sampling sites marked as solid black stars. Bivalves and gastropods are collected from sampling site 1 (Chhasra Formation of Miocene age), larger foraminifers are collected from sampling site 2 (Fulra Limestone Formation of Bartonian age of Eocene) as well as brachiopod and cephalopod specimens are collected from sampling site 3 (Jumara Formation of Callovian age). This map is modified after^47^. (**b**) Outline map of India showing the onshore (Zone-A) and offshore (Zone-B) sampling sites. Inset map (Zone-B) shows the positions of National Gas Hydrate Program Holes 10D, 3B and 5C in the Bay of Bengal. Solid stars represent sampling sites. Planktic foraminiferal specimens were collected from core tops of these sites [drawn on COREL DRAW X7 (Version 17.1.0.572, https://www.corelindia.co.in/index_corel) Software].

The selection of site/location and type of specimens (micro and macro forms), both are crucial for this type of spectral study. It is important to differentiate the effects of diagenetic history and the primary fossil shell geochemistry as diagenetic overprints commonly hide mineralogical attributes of primary shell features^41^. Therefore, the target site should be a depositional setting that can contain well-preserved fossil assemblages with evidence of little to no diagenetic alteration. The larger benthic foraminifera and macrofossils were collected from the pericratonic Kachchh basin, Gujarat, north-west India. The Kachchh basin is one of the most suitable sites for this type of study as this basin accommodates a thick pile of fossiliferous Mesozoic-Cenozoic (middle Jurassic to Recent) sediments with good preservations of marine fauna (Fig. 1a). Besides, one stratigraphic horizon is expected to possess an almost similar diagenetic history. Larger foraminifera specimens (*Alveolina* sp., *Nummulites* sp. and *Discocyclina* sp.) were collected from the stratigraphic horizon of Fulra limestone (Bartonian age, upper Eocene) Formation exposed near Kharai village. These limestones were deposited in a shallow marine environment, restricted from the continental shelf to the slope region^42–44^. The spectral contrast between the larger foraminifera specimens collected from one stratigraphic horizon of Fulra Formation would help in differentiating the ecological and depositional environment signatures as captured and distinguished by reflectance spectra. The bivalve (*Venus* sp.) and gastropod (*Turbo* sp.) fossils were obtained from the highly fossiliferous limestone of Chhasra Formation; (Burdigalian age of Miocene); deposited in the shallow shelf environment^42^. Both of these Formations (Fulra and Chhasra) from the Kachchh basin have signatures of a significant influx of Deccan volcanic basalt^42–44^. The brachiopod (*Kallirhynchia fornix*) and cephalopod (*Hecticoceras* sp.) samples were collected from the Jumara Formation (Callovian age of the Jurassic) and sediments were deposited from a mixed metabasic and felsic source^45^. The Jumara Formation was developed during the rift-climax, much before the Deccan volcanism, in a marine transgressive phase marking the maximum flooding surface^46^, whereas, post-rift Fulra Limestone and Chhasra Formations were developed after the Deccan Volcanism during transgressive phases^46^. Following the aforesaid thematic approach, the spectral contrast between the bivalve-gastropods and the brachiopod-cephalopods respectively would depict their paleoecology and paleoenvironment.

The planktic foraminifera were not compared with the larger benthic forms in terms of their respective spectral features. Planktic foraminifera were rarely observed from the studied larger foraminifera dominated litho-units of Fulra limestone Formation of Kachchh^43^. Well preserved calcitic planktic foraminifera fossils were collected from the microfossil rich Quaternary sediments of Krishna–Godavari (K–G) basin (a pericratonic basin, located along the east coast of India and extended into the Bay of Bengal) at a water depth ~ 1000 m (Fig. 1b, Zone B^48^). Quaternary sediments of the K-G basin also experienced prominent signatures of a significant influx of Deccan basaltic products as it was observed in the Kachchh basin^49^. The purpose of the spectral analysis of planktic foraminifera is to identify the spectral characters which can differentiate the biomineralization strategies between planktic and benthic foraminifera in the environments with enough supply of biominerals like iron (Fe) and aluminum (Al).

## Results and discussions

### Spectral contrast in microfossils

**Figure 2:**
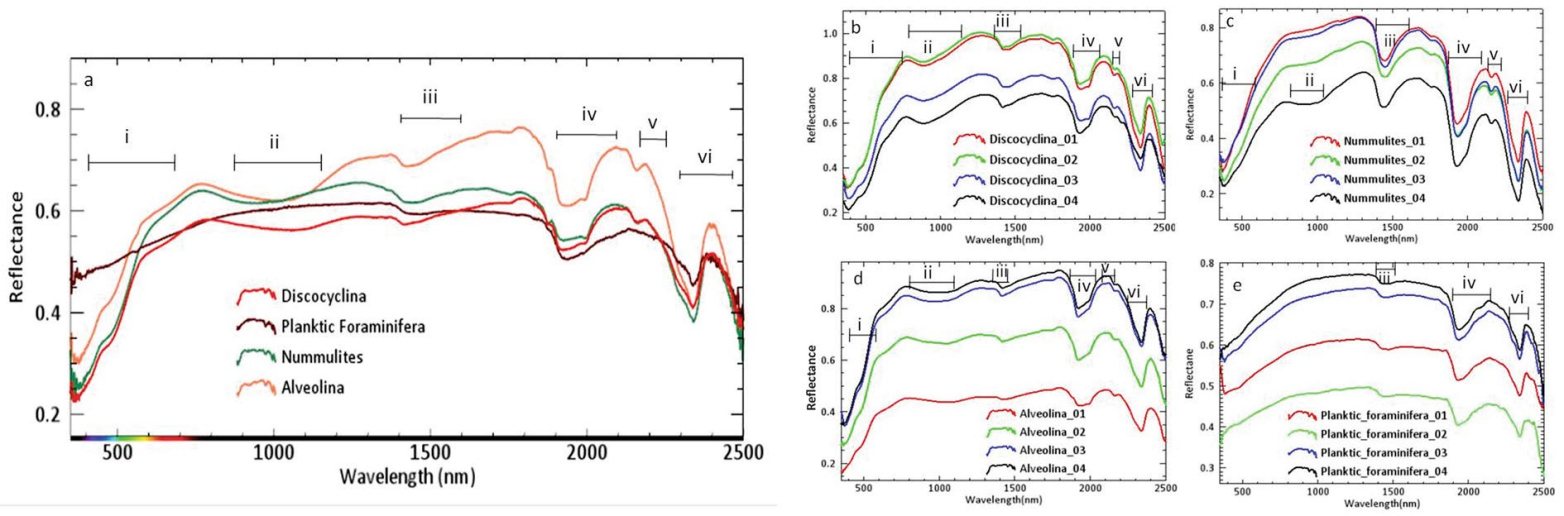
(**a**) Mean reflectance spectra of planktic and benthic foraminifera considered in this study. (**b**–**e**) Reflectance spectra collected from four individuals of each variant of samples of foraminifera. *Discocyclina* (**b**), *Nummulites* (**c**), *Alveolina* (**d**), planktic foraminifera (**e**) are presented to understand the spectral contrast of intra fossil shells. Bars represent i = Fe impurities/coating electronic transition, ii = Fe impurities/coating-crystal field effect, iii = bonded OH molecule-vibrational feature, iv = free OH-vibrational feature (i.e. presence of water), v = Al–OH-vibrational overtone and vi = CO_3_-feature. Planktic foraminifera show different spectral features than benthic foraminifera in terms of Fe and Al–OH fields.

Reflectance spectra of different benthic and planktic microfossils were analyzed (Fig. 2a). Four to five samples from each variant of benthic (*Alveolina*, *Nummulites*, *Discocyclina*) and planktic foraminifera were also analyzed to understand the spectral variation in the different samples of fossil shells of the same group (from benthic foraminifera as well as in planktic foraminifera) if any (Fig. 2b–e). It is important to identify those consistent spectral features (in terms of their wavelength) imprinted on the spectra of the samples of different fossil groups before identifying the spectral feature or spectrometric parameter of a spectral feature (such as depth, width and asymmetry of the feature). These spectral features which are distinct in the spectra of one fossil group regarded as sensitive to biomineralization. Prominent spectral absorption features with their wavelength of absorption minima at ~ 450 nm, 1400 nm, 1900 nm, 2100–2200 nm and ~ 2350 nm in the representative reflectance spectrum of these samples were identified. A broad absorption feature in the spectra of these fossil samples with its absorption minimum at 1100 nm in their reflectance data was also recognized (Fig. 2a). An overview of the spectral features identified on fossil spectra, their importance and their respective wavelength of absorption are discussed below (Table 1). Some of these features are common in the spectra of planktic and benthic foraminifera (e.g., spectral feature at ~ 1400 nm, ~ 1900 nm and ~ 2350 nm), but the width and depth of these features are variable (Fig. 2). The width and depth of the spectral features often indicate the relative abundance of particular mineral constituents^5^. Besides, some absorption features are prominent to one group of foraminifera (~ 450 nm, ~ 1100 nm, ~ 1400 nm, ~ 1900 nm, and 2100–2200 nm features are present strongly in the benthic form).

**Table 1:** Spectral features identified on the studied micro and macrofossils and their importance.

I	II	III	IV	V	VI
450 nm	900–1200 nm	1400 nm	1900 nm	2100–2200 nm	2330–2350 nm
This spectral feature is indicative of the presence of Fe impurities/coating due to the electronic transition. This spectral feature is resulted due to charge transfer process of elements which enter into the fossil shells. So, it is an indicator of primary shell characteristics	This feature is used to distinguish different Fe oxides and hydroxides. The wavelength of the features vary from 900 nm (hematite), 1000 nm (goethite), 1010 nm (limonite) and 1200 nm (siderite). The depth and width of the feature is also important criteria along with the mineral to distinguish different minerals. It is deeper and narrower for hematite and wider for mineral like siderite. The wavelength of absorption minima of the feature shifts from lower to higher wavelength from hematite to goethite. This spectral feature is related to post depositional set-up	The feature is resulted due to the stretching vibration of OH bond. It is a signature of primary shell feature	The feature indicates free H_2_O molecule. The presence of this feature and the depth and width of the feature may indicate the degree of diagenesis happened in the shell (i.e. with better compaction and intense recrystallization, the depth of the feature is reduced)^31,32^. It is a signature of primary shell feature	This feature is indicative of metal-OH vibrational overtone. Presences of clay minerals on the outer shell surface are responsible for these absorption features	This feature is resulted due to vibration of carbonate bond. One of the prominent vibrational features of calcite. It is an indicator of primary shell characteristics

**Figure 3:**
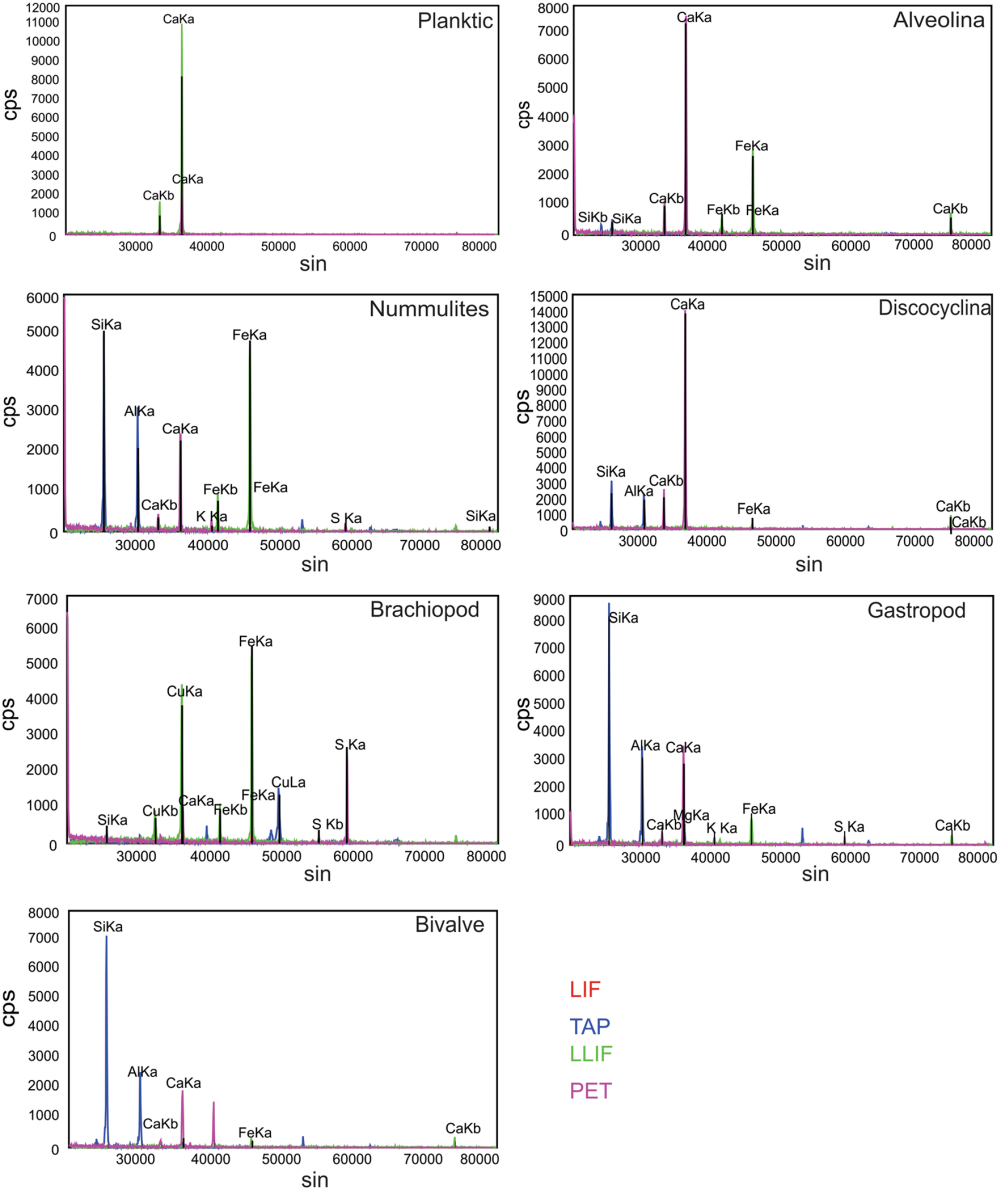
WDS spectra of different foraminiferal (*Alveolina*, *Discocyclina*, *Nummulites*, Planktic) and invertebrate macrofossils (bivalve, brachiopod and gastropod) obtained through EPMA analysis. Fe and Al are absent in planktic foraminifera, whereas merely present in gastropod and bivalve. Enrichment of Fe and Al is observed within the surficial part of *Alveolina*, *Discocyclina*, *Nummulites*, and brachiopod.

**Figure 4:**
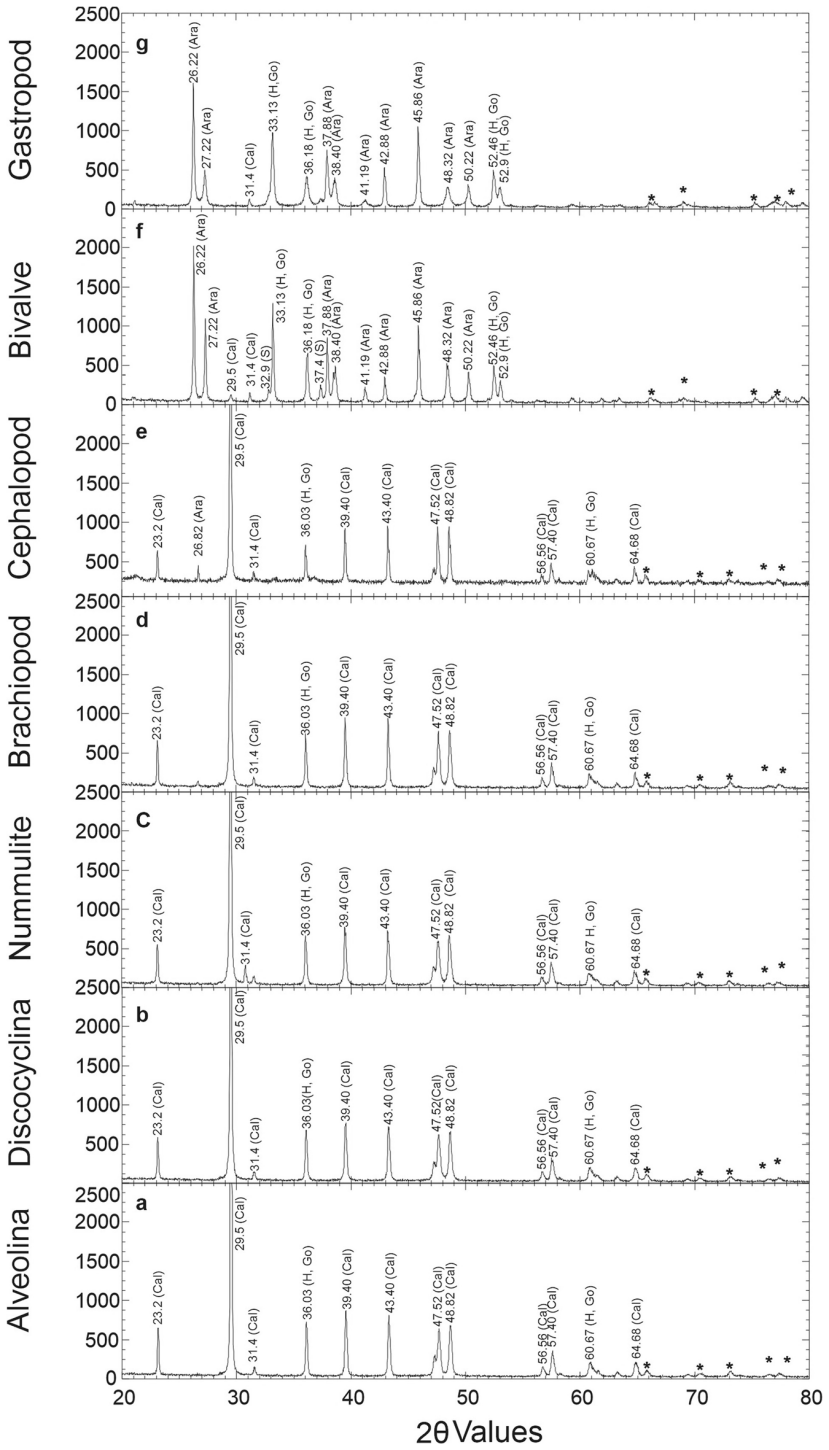
X-ray diffractograms of different specimens of *Alveolina* (**a**), *Discocyclina* (**b**), *Nummulites* (**c**), brachiopod (**d**), cephalopod (**e**), bivalve (**f**) and gastropod (**g**) are presented in this figure. Calcite (Cal) is detected as the main constituent for fossil shells of *Alveolina* to cephalopod (**a**–**e**). Also, two peaks of Fe oxide (Hematite marked by H) and hydroxide (Goethite marked by Go) are detected for the same fossil groups (**a**–**e**), whereas bivalve (**f**) and gastropod (**g**) are consisting of aragonite (Ara). Also, four peaks of Fe oxide (Hematite marked by H) and hydroxide (Goethite marked by Go) are detected for the same fossil groups (**e**,**f**). Minor Siderite peaks (S) are identified as traces in bivalve specimens. Mixed phases of Ca–Al–Fe silicate hydroxide hydrate are present in all the shells and marked by an asterisk (*).

We identified a strong absorption minimum at 450 nm imprinted in the spectra of benthic foraminifera *Alveolina*, *Discocyclina*, and *Nummulites*. This spectral feature results from the electron transition in Fe and indicates Fe impurities/coatings within the shell surface^5,50^. The intensity of this spectral trough (the relative depth of the absorption feature) is much weaker in the case of planktic foraminiferal assemblage. The 1100 nm absorption feature is related to the presence of Fe in the crystal structure (i.e., tetrahedral site) of certain minerals (hematite/goethite or other iron oxides) due to crystal field effect (Fe-oxide coating)^51^ and is prominent in benthic foraminifera. The same absorption feature is absent in planktic foraminifera. Absorption features at 1400 nm and 1900 nm are prominent in benthic foraminifera compared to their planktic counterparts. These features are attributed to OH vibration in the bonded molecule and free water molecule in the sample, respectively^5,51,52^. Finally, the prominent absorption minima at 2350 nm indicates the presence of calcite/dolomite in the carbonate test of both planktic and benthic foraminifera^5,31^. In addition to the above spectral features, few absorption kinks or spectral features are also found on the reflectance spectra of benthic variants within the spectral domain of 2100–2200 nm while these kinks are absent in the planktic foraminifera (Fig. 2a). These features can be attributed to the aluminum silicate impurities because Al–OH vibration processes are responsible for these absorption features^5,31^. Detailed mineralogical analyses of spectral features show contrasts between the specimens of different species of the larger benthic foraminifera (Table 2, Fig. 2b–d). All the *Discocyclina* specimens have signatures of hematite, whereas the *Nummulites* and *Alveolina* specimens are coated with mostly goethite (Table 2, Fig. 2b–d). The Wavelength Dispersive X-ray Spectroscopy (WDS), Spectra of Electron Probe Micro Analyzer (EPMA) (Fig. 3) and X-ray diffractogram (XRD) (Fig. 4) also confirm the presence of Fe, Al and their mineral phases (Fe–OH hydroxide, oxides and Al–OH hydroxide) respectively in all benthic foraminifera along with calcium carbonate. The shallow water (shelf environment) bottom-dwelling nature of benthic foraminifera may have attributed these absorption features. These features are absent in the planktic foraminifera. The enrichment of Fe, Al and their mineral phases within the benthic tests can be attributed to diagenesis, presence of surficial clay minerals, or to biomineralization. Some secondary materials (Fe, Al and their mineral phases) may also be incorporated during diagenesis within the benthic forms. However, the larger benthic foraminifera are resistant to diagenetic alteration due to their test morphology (especially wall structures)^35,53^. The studied larger benthic foraminiferal samples are also free from any diagenetic alteration (Supplementary Fig. S1). Besides, the impact of diagenesis is expected to be same in all the samples of each group of benthic foraminifera as these fossil samples are collected from the same Formation (biostratigraphic unit). So, there are two possible reasons for this specific mineral affinity of benthic foraminiferal species within the same diagenetic setting. Firstly, the modest spectral contrasts between the benthic forms may indicate their contrasting preference to foods, i.e., novel biomineralization strategy that leads to different shell chemistry, which works with a favorable substrate for different Fe-oxide precipitation^35,54,55^ (Fig. 2b–e). Lastly, micro seasonality and small scale climatic-environmental changes may also attribute different types of Fe-oxide/hydroxide coatings (hematite can form in a warm and dry climate whereas goethite form in more wet condition) on shells within a small period of time^54^. Hence, the genesis of the Fe-oxides and hydroxides and their complex relationship with fossil substrate require extensive and rigorous studies^54^.

**Table 2:** Mineralogical analysis of spectral feature of different microfossils.

Benthic foraminifera			Planktic foraminifera
Discocylcina	Alveolina	Nummulites	
The presence of absorption feature in 880 nm indicative of hematite (labeled as II in Fig. 2a,b). The absorption feature due to H_2_O is also wider and deeper (labeled as IV in Fig. 2a,b). This indicates that the fossil shell had minimal imprint of digenetic compaction and recrystallization^31,32^ Small absorption kink (resulted due to combined effect of vibration of AL–OH bond and C–O bond) at 2200 nm are present. This feature may indicate clay minerals as clay is also reported in FESEM	The absorption feature approximately at 990 nm (II in Fig. 2a,d). The feature indicative of goethite. In addition to that the spectral feature resulted from Al–OH bond (V in Fig. 2a,d) and C–O bonds (VI in Fig. 2a,d) are also present. The depth and width of the spectral feature indicate the presence of free H_2_O with good quantity and this indicate minimal imprint of digenetic compaction and recrystallization^31,32^. Absorption kink at 2200 nm indicates the presence of surficial clay minerals and supplemented by FESEM analysis	Nummulites have similar spectral feature as the feature observed (Fig. 2c) in alveolina but spectral feature with wavelength of absorption at around 900–1000 nm is weaker; indicate presence of goethite but as trace. The depth and width of the spectral feature is intense and it indicates the presence of free H_2_O with good quantity. This in turn indicate minimal imprint of digenetic compaction and recrystallization^31,32^. Spectral feature at 2200 nm is present. The presence of clay minerals is supported by FESEM analysis	The spectral feature at 450 nm and 900–1000 nm are absent. Strong presence of carbonate feature (VI in Fig. 2e) and wider and deeper spectral feature (IV in Fig. 2e) indicative of minimal imprint of digenetic compaction and recrystallization^31,32^. No spectral feature at 2200 nm (V in Fig. 2e) is present

**Figure 5:**
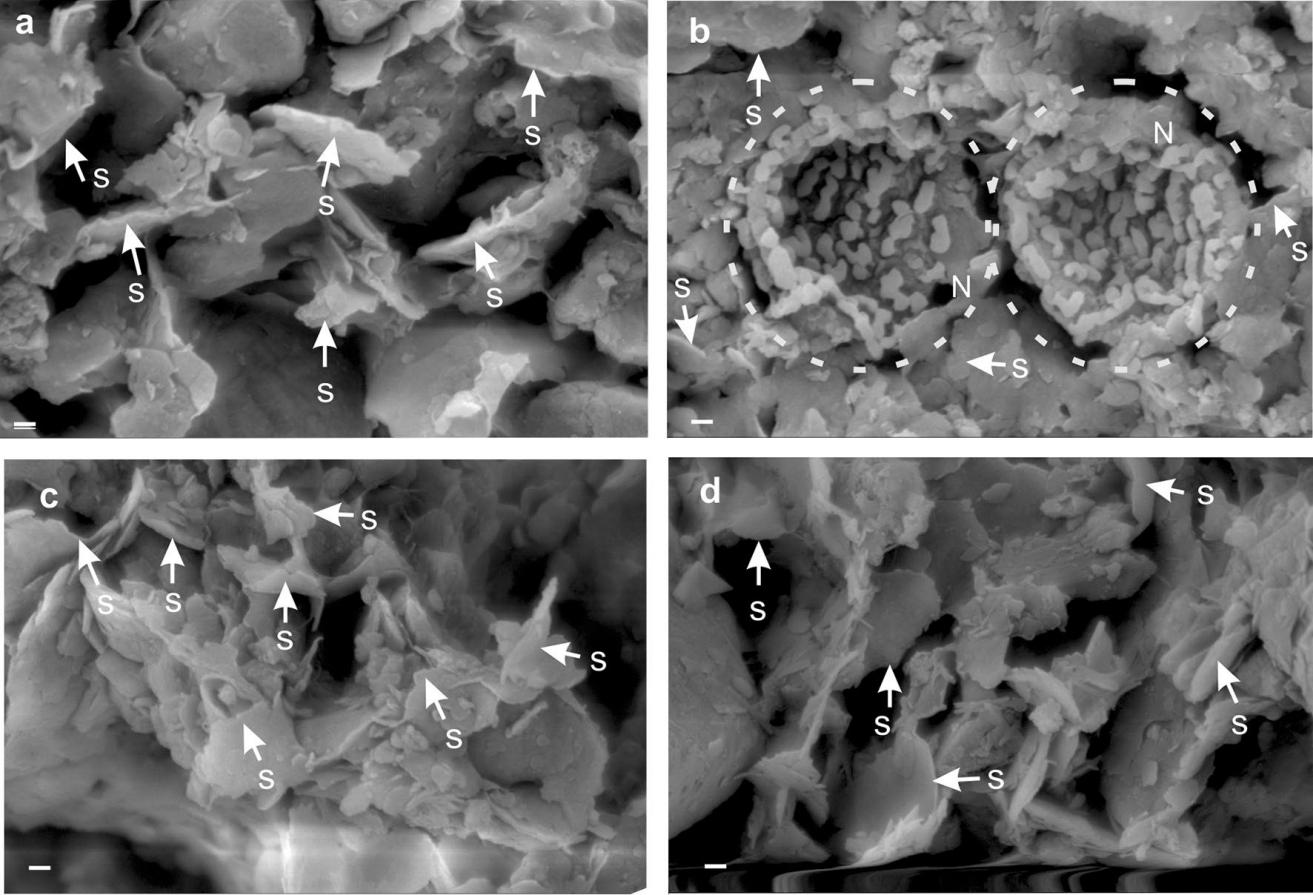
SEM images of surficial clay minerals collected from the outer part of fossils showing the presence of smectite (indicated by white arrows) and nontronite (encircled by the white dotted line). The identification of clay minerals was made based on the published catalog^56^. (**a**) Smectite (S) present on *Alveolina* test (**b**) Nontronite (N) and Smectite (S) present on *Nummulites* test (**c**) Smectite (S) on bivalve shell (**d**) Smectite (S) on gastropod shell. All the scale bars represent 200 nm.

In a nutshell, ~ 450 nm, ~ 1400 nm, ~ 1900 nm, and ~ 2300 nm features observed in the spectra of microfossils are indicative of primary shell characteristics, whereas 900–1200 nm, and 2100–2200 nm features exhibit the depositional history of the site. A strong absorption feature around 1100 nm resulted due to crystal field effects for the presence of Fe-oxide and absorption feature at 2200 nm due to molecular vibration of the Al–OH bond are some of the spectral keys for identifying few important variants of benthic foraminifera and also to distinguish them from planktic varieties. This observation is also supplemented by EPMA analysis, which indicated the concentration of iron (Fe), aluminum (Al) and their mineral phases to be much depleted in planktic forms in comparison to their benthic counterpart. The Field Emission Scanning Electron Microscope (FESEM) images helped to identify the presence of a traceable amount of smectite ([(1/2 Ca, Na)_0.7_ (Al, Mg, Fe)_4_ [(Si, Al)_8_O_20_] (OH)_4_*nH_2_O]) and nontronite ({(Na_Tr_1/2Ca)_0.46_(Al_0.05_Fe_1.93_Mg_0.12_) [Al_0.5_Si_3.5_O_16_(OH)_2_]}) on the surficial part of some benthic specimens (Fig. 5) whereas planktic foraminiferal tests are bereft of any kind of clay minerals. The clay minerals present in the benthic shells were considered to be responsible for introducing vibrational overtone absorption features of the Al–OH bond (Fig. 2a).

Studies revealed that few elements (Fe, Al) might be incorporated in the foraminiferal tests during biomineralization^25,26,57,58^. Biomineralization is a process by which the living organisms synthesize foreign inorganic elements in their test during the shell forming process^25,26,57,58^. Different organisms take different biomineralization strategies to form their shells. This is diversified from species to species^55^. Iron is considered as an essential micronutrient for phytoplankton and is used in different biogeochemical processes^59^. Also, Fe is almost insoluble in oxic water. Therefore, the mixed layer water contains less Fe owing to its consumption by phytoplankton under the presence of the well-oxygenated environment. This could be the possible cause of Fe enrichment in the benthic fossil shells. Moreover, the Al content is always higher in the shelf environment (close to the continental margin) due to the deposition of terrestrially derived minerals^60,61^. So, the presence of the above minerals is recorded on the reflectance spectra of different foraminifera and it reveals the depositional history of the basin and paleoecology.

### Spectral contrast in macrofossils

**Figure 6:**
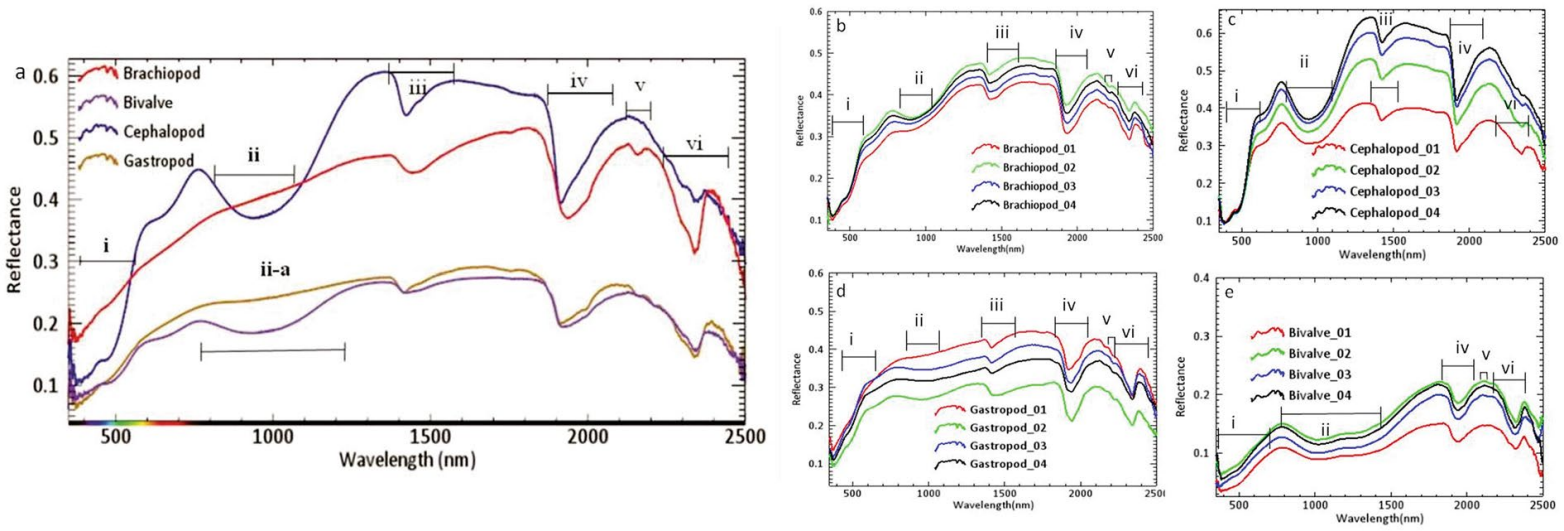
(**a**) Mean reflectance spectra of various macrofossils (gastropod, bivalve, brachiopod, and cephalopod) considered in this study. Wider range of spectra (at 1030 nm) is observed only in bivalves indicate the presence of siderite (marked by ii-**a**). (**b**–**e**) Reflectance spectra of samples of each variant of studied macrofossils of brachiopod (**b**), cephalopod (**c**), gastropod (**d**), and bivalve (**e**) are presented to understand the spectral contrast of intra fossil shells. Bars represent i = Fe impurities/coating electronic transition, ii = Fe impurities/coating–crystal field effect, iii = bonded OH molecule-vibrational feature, iv = free OH-vibrational feature (i.e. presence of water), v = Al–OH-vibrational overtone and vi = CO_3_-feature.

It is important to analyze the spectra of different samples of the same macrofossil group or phyla (Mollusca) and also the representative reflectance spectra of different phyla (Brachiopoda) (Fig. 6a). This is necessary because due to their wide range of aquatic life habitats and their potential in preserving significant geochemical signatures that are indicative of these habitats despite having significant diagenetic alteration^62^. In this regard, four samples of each macrofossil variants were analyzed. It was confirmed that the different samples of each variant of phyla were mineralogically similar. This is evident from the fact that the wavelength of the diagnostic spectral features of the samples from the same fossil phyla remains identical. We also made a comparative analysis of the representative spectrum of four macrofossils of different classes (Table 3). Few samples of each variant of macrofossils were also studied to understand the spectral contrast between the samples of the same fossil group (Fig. 6b–e). However, each fossil group is spectrally distinct from the other. We found reflectance spectra of gastropods and bivalves are similar in terms of the presence of absorption features at a specific wavelength (Fig. 6a). The only difference being bivalves have absorption features at 1030 nm (marked by ‘ii-a’ in Fig. 6a, ‘ii’ in Fig. 6e), which is probably due to the presence of siderite. However, XRD and other mineralogical data could confirm the presence of siderite as traces. Cephalopods showed a completely different spectral behavior. A broad absorption feature at 950 nm was prominent only in the cephalopod fossils and this spectral feature is due to iron hydroxides (Fig. 6a,c). FESEM investigation reveals the presence of smectite in all the specimens except cephalopods. Cephalopod specimens were encrusted with yellowish-brown color sediments considered as limonite [FeO(OH), nH_2_O] distinguished through optical properties under a polarizing microscope. Therefore, the spectral trough for Fe enrichment in cephalopod (marked as ii in Fig. 6a,c) is linked to the crystal field effect due to the presence of Fe in the crystal field structure of limonite. Goethite has crystal field absorption features at a relatively lower wavelength than limonite (a few nanometers apart from each other). Brachiopods have prominent goethitic features (900–1000 nm) and strong Al–OH features at 2100 nm instead (Fig. 6a,b). XRD data of all these specimens confirm the presence of Fe–OH in their shells (Fig. 4). EPMA study showed the presence of FeO within the shells of these macro faunas (Fig. 3).

**Table 3:** Mineralogical analysis of spectral feature of different macrofossils.

Gastropod	Bivalve	Cephalopod	Brachiopod
Gastropod has weaker spectral feature at 900–1000 nm wavelength (ii in Fig. 6a,d). This indicates the presence of Fe–OH but as trace. Other spectral features are similar with the features identified in other macrofossils (Fig. 6a,d). This is supported by other mineralogical data. Spectral signatures of minimal diagenetic alteration are present	The spectral feature with the wavelength of absorption minima at 1030–1200 nm (ii-a Fig. 6a, ii in Fig. 6e) has wider range and this is may be due to the presence of siderite. However, XRD and other mineralogical data confirm the presence of siderite as traces. Spectral signature indicates that the fossil shells have minimal imprint of digenetic compaction and recrystallization. Other primary features (i, iv, vi in Fig. 6e) are prominent	The cephalopod has narrow spectral feature with absorption minima at 1010 nm; it is indicative of the presence of Fe-hydroxides. It shows inconspicuous 2300 nm Al–OH vibrational feature (Fig. 6c). No such clay minerals are observed by other geochemical analysis. Spectral signatures indicate the shells are free of diagenetic alteration	Brachiopod has asymmetric spectral feature at 900–1000 nm (ii Fig. 6a,b). This indicates the presence of Fe–OH. Al–OH vibrational feature (V) is more prominent in brachiopod than cephalopod. Presence of surficial clay mineral is observed by geochemical analysis. Spectral signatures of minimal diagenetic alteration with primary shell features are present

These invertebrates incorporate Fe and Al from habitat in their shells during biomineralization^63^. Gastropods mostly live in inner neritic to shallow shelf environment^64,65^. Bivalves also physically prefer the same habitat and show a common association with gastropods. The bivalve and gastropod samples were collected from the limestone layer of Chhasra Formation, deposited in a shallow marginal to inner shelf condition^42^. The only difference is that the bivalves are mostly burrowers, while the gastropods are surface dwellers. Furthermore, brachiopods are a relatively deep-water dweller, mostly living within the shelf environment, while nektonic cephalopods are found at greater water depths^36,62^. The spectral profiles of gastropods, brachiopods, and bivalves are alike in terms of the shape of the reflectance spectra and the presence of spectral features at the same wavelength (Fig. 6a). However, the cephalopods reveal different spectral features from the other three macrofossils (prominent Fe-crystal field feature at 950 nm and inconspicuous 2300 nm Al–OH vibrational feature). This may be due to the differences in habitat and feeding habits. Almost all of the bivalves, gastropods, and brachiopods show evidence of a sedentary and solitary lifestyle. They collect their food via filter feeding techniques, whereas cephalopods are mostly vagile/free mover predators^62^. Moreover, bivalves, brachiopods, and gastropods prefer to live in an ambient environment, whereas cephalopods have a record for vertical and/or lateral migrations along with the seawater. So, cephalopods exhibit very different and complex geochemical patterns in their hard shells as they move through different physicochemical properties of different water masses^62^. These ecological preferences may contribute to this spectral contrast, and a detailed study is required.

While analyzing the reflectance spectra of macrofossils, it has been realized that ecological choices might be a vital factor for the incorporation of biomineralized Fe, Al and their respective mineral phases in the macrofossil shells. The presence of imprinted Fe crystal field features in the macro invertebrate fossil shells may be related to post-depositional diagenesis related to depositional settings which results Fe oxides encrustation. Spectrometric parameters such as width and depth of spectral feature at 1900 nm indicate the amount of water present in the fossils^32^. The prominent feature (in terms of depth and width) at 1900 nm indicates the presence of water in the shells and it reiterates the less effective post-depositional diagenetic events^31^. The width and depth of specific absorption features are sensitive to the relative abundance of the element available in the target, responsible for imprinting definite spectral features^66^.

### An overview on the understanding depositional history and paleoecology through reflectance spectroscopy

Spectral signatures of different fossils provide a broad overview of the paleoenvironment with some indications on the paleoecology. Reflectance spectroscopy of the larger benthic foraminifera shows good preservation of Fe, Al, and their oxide-hydroxides in their shells. One of the possible reasons for the abundance of these elements could be bloom in the phytoplankton population due to the availability of micro-nutrients like Fe^67^. Here, the phytoplankton bloom makes a symbiotic relationship with larger benthic foraminifera and helps them to increase their population rapidly^67^. Secondly, the presence of basalt (mainly tholeiitic and enriched in Al_2_O_3_, CaO, FeO and TiO_2_) could also be a potential factor for assimilation of Fe and Al in the benthic foraminiferal test in the Fulra limestone sediments^44,68^. The Deccan basalts derived elements might have been biomineralized in the shells of the benthic foraminifera. Primary shell character and post-depositional diagenetic histories (presence of hematite/goethite and clay minerals) are imprinted on benthic foraminifera’s shell and captured by reflectance spectra. This conforms to the previous studies depicting the Fulra limestone deposited in a warm, tropical, inner ramp to mid ramp settings^43,44^. The spectral contrast between the benthic foraminifera groups may be regarded as the indication of their respective paleoecological preferences^35^. On the other hand, the collected planktic foraminifera are from Quaternary sediments of the open Indian Ocean, away from the coast. Previous studies documented the significant contribution from the Deccan basalt (similar kind of mineral assimilation set up as we observed with the Fulra Limestone, Kachchh) and indication of various diagenetic mineral (like Fe and Al) imprints in the Quaternary sediments of the studied site in the Indian Ocean are also reported^49^. Due to the free-floating habitat and different shell forming process of the planktic foraminifera^40^, the signatures related to Fe and Al were almost missing in their respective spectra. So, the absence of the above features may provide information on their (planktic foraminifera) paleoecology. Furthermore, the biominerals might have been assimilated from weathered basaltic products in the macrofossil shells (bivalve-gastropods and cephalopod-brachiopods) and were influenced by their ecological preferences^42,45^. The spectral contrast between bivalves and gastropods (similarly between brachiopods and cephalopods) deposited in a similar diagenetic and depositional set-up may provide valuable insight on their paleoecology.

Diagenetic processes, like recrystallization, cause the conversion from aragonite crystals structure to calcite. Almost all of the mollusks go through recrystallization and change in their calcitic shell (aragonite to calcite). Though their carbonate shells undergo recrystallization with fossilization processes, biomineralization signatures remain unchanged over the years^69^. So, the original information (related to biominerals) of the fossils collected by reflectance spectroscopy remains unaffected. Besides, reflectance spectra of carbonate minerals like aragonite, calcite, and dolomite are characterized with seven absorption bands (with corresponding spectral features) in the spectral region from 1600 to 2550 nm, imprinted by vibrations of the carbonate radical^70^. Among these absorption bands, spectral feature or absorption band at 2330 mm is prominent for the carbonate minerals. The wavelength of absorption of aragonite and calcite is very close to each other (although they are few nanometers apart) and often will be indistinguishable from each other if both the minerals are present on the fossil surface and remain mixed with other minerals^71^. Retrieving mineralogical information from spectral mixtures is a challenge for spectrally close targets and different spectral decomposition are to be implemented to retrieve spectral end members^71^. The crystallization effect due to the recrystallization of carbonate minerals in benthic macrofossils, as mentioned above may be remained subdued in the respective reflectance spectra of fossils in comparison to the spectral features imprinted due to the crystal field effects and electronic transition processes of different transitional elements which enter into the fossil shells. These elements, such as Mn and Fe, have their spectral features at a different wavelength, and these features would be easily distinguishable^5^. Similarly, different metal-hydroxyl bond vibrational features are also well separable from the vibrational feature of carbonate bonds. These spectral features are often influenced by the depositional surface as well as the depositional medium as discussed earlier.

Reflectance spectroscopy appears to be a very promising tool for rapidly bringing out mineralogical detail of fossils. These data are also found to be consistent with the mineralogical data derived using destructive, time consuming, cost-ineffective analytical methods. Therefore, reflectance spectroscopy can be used as the key analytical method for analyzing mineralogical details of a large volume of fossil samples to understand the paleoenvironment and diagenesis of samples across different parts of the larger basin. Future effort can also be made to identify the spectral contrast between fossil-bearing and fossil depleted host rock to understand the possibility of using the imaging spectrometer data in detecting fossiliferous lithological horizons. Conjugate analysis of spectral features of rocks and fossils would also help to identify spectral features indicative of biomineralization in a more affirmative manner. This spectral contrast can be helpful in targeting and mapping fossil-bearing carbonates using remote spectrometer from the airborne or space-borne platform. Reflectance spectroscopy provides a good alternative to fossil studies with the conventional time consuming costly geochemical methods and can make the fossil findings much easier during the field survey.

## Conclusions

Non-destructive, rapid natures of analysis and near-perfect measurements make the reflectance spectroscopy a useful and innovative tool for determining the paleoenvironment of a sedimentary basin. It can be utilized as a potential tool for prelude investigations of various fossils before taking up detailed biological and geochemical analyses. The present study concludes that:

- Reflectance spectroscopy derives important mineralogical information for the micro and macrofossils, which complements the mineralogical data derived from EPMA, FESEM, and XRD data. This indicates the potential utility of reflectance spectroscopy as an alternative analytical technique. As it is also a rapid and non-destructive method, reflectance spectroscopy may take a lead role in the mineralogical analysis of large volumes of samples to infer the basin evolution process.
- Reflectance spectroscopy could delineate mineralogical contrast between different benthic foraminiferal species. Spectral features at 450 nm (Fe-oxide electronic transition), 900 nm (Fe-oxide),1100 nm (Fe coating/crystal field effect due to Fe–OH), 1400 nm (OH vibration in the bonded molecule), 1900 nm (free water molecule), and 2100–2200 nm (Al–OH vibration) were prominent in benthic shells.
- The spectra of planktic foraminifera assemblage was characterized by ~ 2350 nm (CO_3_ features). The spectral feature at 450 nm is also weak in planktic foraminifera. Ecological behavior, feeding pattern, habitat, and shell mineralogy/diagenesis of macrofossils often control the reflectance spectra as variation in the delineated minerals can be attributed due to contrasting paleoenvironment and different paleoecological behavior. Planktic foraminifera take up an insignificant amount of Fe and Al, whereas benthic forms assimilate more Fe and Al in their shells during biomineralization. The paleoecological attributes can be analyzed from the fossil samples of different groups that proliferated under the same paleoenvironment if their spectra have contrasting spectral features.
- The spectral features at ~ 450 nm, ~ 1900 nm, 1400 nm and ~ 2300 nm features are indicative of primary shell characters that define ecology. On the other hand, the 900–1200 nm, and 2100–2200 nm features exhibit the depositional history of the site.
- Similarly, mineralogical differences of macrofossils are also well defined by their respective reflectance spectra. The Fe crystal field feature at 900 nm is prominently observed only in the cephalopod and spectral features at 1030 nm is identified in the spectra of bivalve fossils. The 2300 nm Al–OH vibrational overtone feature is absent in cephalopod fossils. These features may give valuable insight to paleoecological behavior and their paleoenvironment.

## Materials and methods

### Materials

We used multiple numbers of different foraminiferal (larger benthic and planktic) and invertebrate (brachiopod, bivalve, gastropod, and cephalopod) fossils in this study. Benthic and planktic foraminifera, along with different macrofossils, were chosen for this study as all these forms were marine and preferred to live within a similar mixed layer zone/shelf environment. While the benthic forms are stable at different depths, the surface dweller (planktic foraminifera) and nektonic (cephalopod) forms represent surface water as well as mid-depth water geochemistry. Larger benthic foraminiferal specimens (*Alveolina* sp., *Nummulites* sp. and *Discocyclina* sp.) were varying in size and ranged between 5 and 20 mm. Planktic foraminiferal fossils (size range > 125 microns) were collected from different National Gas Hydrate Program Holes (10D, 3B and 5C) from the Quaternary sediments of Bay of Bengal, Indian Ocean. They mainly consisted of species like *Globorotalia menardii*, *Globigerinoides ruber*, *Globigerinoides conglobatus*, *Gs. sacculifer*, *Gs. quadrilobatus*, *Orbulina universa*, *Neogloboquadrina dutertrei* and *Sphaeroidinella dehiscens*. However, planktic foraminifera were grouped as an assemblage and their integrated reflectance spectrum was collected.

In addition, to the use of foraminifera, we also used representative samples of invertebrate fossils, which belong to Phylum Brachiopoda (*Kallirhynchia fornix,* middle Callovian), Phylum Mollusca of Class Cephalopoda (*Hecticoceras* sp., upper Bathonian to lower Oxfordian), Class Bivalvia (*Venus* sp. belongs to superfamily Veneracea, Miocene) and Class Gastropoda (*Turbo* sp., Miocene) for spectral analysis. The selection of the sample site is the most important part of this study to ensure that all the samples had a similar depositional history with good preservation of shell structures of the fossil specimens. Light microscopic observation and SEM study of the different studied micro and macrofossils show that shells of the fossils are preserved with the prominent presence of all the wall structures and free of diagenetic effect (Supplementary Fig. S1). However, a lower degree of diagenetic effects is observed in the shells of bivalve with almost intact primary structures (Supplementary Fig. S1).

### Methods

#### Reflectance spectroscopy

We collected spectral profiles of the samples under laboratory conditions. The planktic foraminiferal assemblage was placed on a sample holder covered with black paper (4 in. × 3 in.). Reflectance spectra were collected for the samples using optical fiber (i.e., measurement optics) of FIELDSPEC 3 spectroradiometer having a field view of 25°. Sample to fiber-optic distance was kept 1–2 cm to derive measurement spots ranging from 0.2 to 0.4 cm radius^72^. The samples were irradiated using a halogen lamp. Spectral radiance of fossil samples has been normalized using the radiance of the white Lambertian plate (which is used to estimate the incoming radiance from the illuminator assuming the plate radiates whatever light falls on it) to derive reflectance of fossil samples. The spectrometer used in this study is operative within the spectral domain of 350–2500 nm reflectance. The approach followed in the study on the details of sample preparation, collection procedures of spectral profiles under laboratory conditions, analysis of these spectra have been discussed in previous literature^15,73^.

To analyze the spectral signature of intrafossil group samples, spectral data collection and analysis for four to five specimens of each group were made. We collected spectra for five to six sample spots for each foraminiferal sample (i.e., sample box), and these spectra were averaged to derive the mean spectra of each sample. Subsequently, averaging of spectra samples of each group would derive representative spectra of each micro and macrofossil type.

#### Powder X-ray diffraction (XRD) analysis

XRD study is a destructive analytical technique used for characterizing crystalline samples, identification of various mineral phases. XRD was carried out to identify minerals associated with the hard shells of the studied fossils for relating spectral features with the mineralogy. For this purpose, we followed the sample preparation and analysis procedures described previously^74^. We used the outermost part of each invertebrate fossil and foraminiferal specimen for XRD analysis. Around 2 g of the finely powdered sample (230 mesh) of fossils was used. Then, the powdered sample was placed in the sample box and irradiated with X-ray. XRD analysis was performed in X'PERT PRO XRD machine with Cu Kα target, equipped with a fast x'Celerator solid-state detector. The XRD scanning range from 20° to 60° is optimum for the characterization of carbonate rock samples^75^. However, we used an extended range (4°–80°) for this purpose. Identification of different mineral phases was made using the Joint Committee on Powder Diffraction Standards (JCPDS) data cards.

#### Field Emission scanning electron microscope (FESEM) and light microscope

FESEM imaging is a swift and robust technique for morphological studies. This technique has widely been used for the identification of clay minerals^56^. Images are produced by the interaction of the samples with primary electron beams that identify any microscopic objects (up to nanometer scale) such as clay minerals, grain shapes, wall structures of organisms, or for topographic characterization of any micro size particles^56^. For this study, FESEM analysis was conducted along with the XRD analysis for the identification of attached clay minerals on the outer part of the shells^56^. Clay materials were separated from outer shells during ultrasonic cleaning. The sample preparation technique involved various steps like sample stubbing, mounting and imaging. FESEM imaging of the uncoated clay mineral sample was conducted by ZEISS SUPRA 55 under laboratory conditions. We used the published catalog^56^ for clay mineral identification. Microstructure studies of the different fossil specimens were done in the same FESEM. Light microscopic observation was performed under the Zeiss Axio Imager M2m microscope.

#### Wavelength dispersive X-ray spectroscopy in electron probe micro analyzer (EPMA-WDS)

EPMA study is an efficient technique for the qualitative and quantitative study of any mineral within a specified point or area^76^. Samples are bombarded with electron beams and sample-electron interactions emit backscattered electron and X-rays^77^. Though the imaging procedure of EPMA is very much related to SEM, yet it gives detailed insight about major and other trace elements present within the fossil shells through attached WDS and could be supportive evidence coupled with XRD and FESEM observations. The sample preparation method for EPMA study is similar to the procedures followed in FESEM. In the laboratory, multi-spectrometer elemental oxide analysis was done with the CAMECA SX5 EMPA instrument with high voltage and 15nA current conditions. Multiple elements (Fe, Si, Al, Ca, Cu, K, S) were selected for the quantitative analysis. We used WDS analysis for reconfirmation of different elements present in the sample. Very High spatial resolution of WDS allows finding out very small chemical zonings and minimal chemical phases. ZAF (Z = atomic number correction; A = absorption correction; F = characteristic fluorescence correction) corrections were used to remove the interelement (matrix) effects.

## Supplementary information

Supplementary Figure S1.
