# Supplementary material for: Potential utility of reflectance spectroscopy in understanding the paleoecology and depositional history of different fossils
Source: Sci Rep. 2020 Oct 8;10:16801. doi: 10.1038/s41598-020-73719-4 (PMC7545181; doi:10.1038/s41598-020-73719-4)
Supplement: Supplementary file 1 — Supplementary Figure S1. [file 41598_2020_73719_MOESM1_ESM.pdf]

Potential Utility of Reflectance Spectroscopy in Understanding the Paleoecology and Depositional History of Different Fossils

Swagata Chaudhuri[1*], Arindam Guha[2], Ajoy K. Bhaumik[1], Komal Pasricha[3]

[1]Department of Applied Geology, Indian Institute of Technology (Indian School of Mines), Dhanbad, Jharkhand-826 004, India

[2]Geosciences Group, National Remote Sensing Centre, Indian Space Research Organisation, Balanagar, Hyderabad-500037

[3]Ministry of Earth Science, Government of India, New Delhi – 110003

*Corresponding author: swagatachaudhuri94@gmail.com

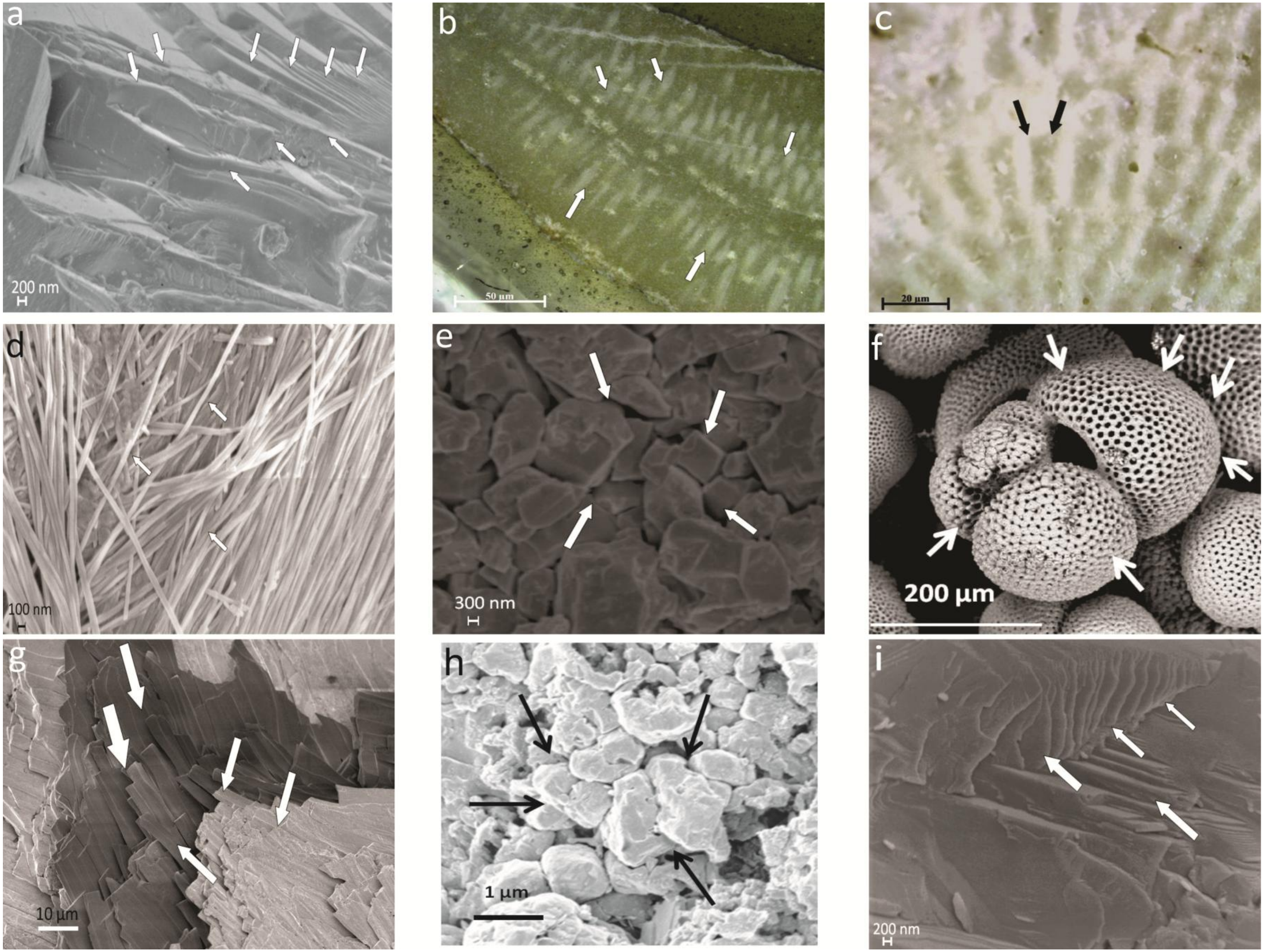
a
200 nm
b
50 µm
c
20 µm
d
100 nm
e
300 nm
f
200 µm
g
10 µm
h
1 µm
i
200 nm

Figure S1: SEM analysis and light microscopic imagery of the different studied micro and macrofossils show that shells of the fossils are preserved with the prominent presence of all the wall structures and free of diagenetic effect (a) SEM imagery of the *Discocyclina* test exhibiting stacks of crystalline calcites in the test, (b) Light microscopic imagery of equatorial sectional view of *Discocyclina* sp. showing lateral chamber arrangement, (C) light microscopic imagery of surface view of *Nummulites* sp. showing whorls and septal filaments, (d) SEM close up exhibiting canaliculated calcitic layers that forms the septal filaments of *Nummulites* sp., (e) Euhedral crystals of calcite inside the *Alveolina* test shown by SEM imagery, (f) SEM imagery of planktic foraminifera showing prominent wall and primary structures and free of diagenetic alteration, (g) SEM micrographs of brachiopod shells showing calcitic fibers which are an indication of primary shell feature and non-diagenesis, (h) SEM micrograph showing granular prismatic tablets of aragonites of the outer shell layers of the bivalve species indicating primary shell features, (i) SEM imagery of the cephalopod shells exhibiting vertical stacks of nacre tablets and nacre preservation. These show the specimens are well preserved; no such diagenetic alteration and recrystallization are recorded.
